# ‘Diabetes Makes You Lose Your Leg’: Footcare Self‐Management Amongst I‐Taukei Fijians—A Wearable Camera Study

**DOI:** 10.1002/hpja.70076

**Published:** 2025-07-10

**Authors:** Keresi Rokorua Bako, Masoud Mohammadnezhad, Dianne Sika‐Paotonu, Amanda D'Souza, Louise Signal

**Affiliations:** ^1^ Health Promotion & Policy Research Unit, Department of Public Health University of Otago Wellington New Zealand; ^2^ Faculty of Health, Education and Life Sciences Birmingham City University Birmingham UK; ^3^ Dean's Department Wellington, Division of Health Sciences University of Otago Wellington New Zealand

**Keywords:** diabetes, Fiji, foot amputations, footcare, self‐management

## Abstract

**Issue Addressed:**

Footcare is an integral part of diabetes self‐management, yet there is limited research on diabetes footcare self‐management. This paper explores footcare self‐management and the impact of diabetes foot complications amongst I‐Taukei Fijians in Fiji.

**Methods:**

This mixed‐method research examines data from the innovative Fijian Diabetes Cam study, consisting of photographic images, photo‐elicitation interviews, and researcher observation. A strategic sample of 30 I‐Taukei Fijian adults with diabetes participated. Participants wore a camera that photographed their activities, behaviour, and environment every 7 s for 4 days. Images were used in semi‐structured photo‐elicitation interviews at home, with the images as prompts. Image data and observation were descriptively analysed, while photo‐elicitation responses were thematically analysed.

**Results:**

While all participants said they performed footcare, for most this was limited to looking for cuts and wounds. Those with foot complications such as amputations have to leave employment. Facilitators included family and community support. Key barriers included the lack of preventive health services and support; poor resourcing and accessibility of dressing supplies, blood sugar testing kits and mobility aids and the warm climate hindering the wearing of closed shoes. Access to appropriate housing and transportation were also key barriers for those with amputations.

**Conclusions:**

This study suggests that I‐Taukei patients recognise the need for footcare self‐management and are largely motivated to do it. However, there is a lack of health education and the health service lacks the resources and trained staff to meet the needs of the people. The underfunded and under‐resourced health system is a major contributor. Nevertheless, there are opportunities to improve health promotion actions.

**So What?:**

Health promotion approach to diabetes footcare self‐management in Fiji could significantly improve the lives of people living with diabetes and reduce the risk of complications and amputation. Donor countries and WHO need to consider the support provided to assist Fiji to manage this increasingly challenging public health issue.

## 1 Introduction

Diabetes is rising rapidly, with more than 1.31 billion people worldwide predicted to be living with the disease by 2050, an increase from 529 million in 2021 [1]. This rise is largely due to the increase in Type 2 Diabetes Mellitus (T2DM), which accounts for 90% of total diabetes prevalence [1, 2]. The greatest relative increase in the prevalence of diabetes between 2021 and 2045 is expected to occur in middle‐income countries (21.1%) compared to high (12.2%) and low‐income (11.9%) countries [3]. In the Western Pacific region, diabetes prevalence is a growing burden, yet there is limited information available on disease trends [4]. In Fiji, a middle‐income country (MIC), 17.7% of adults live with diabetes, and in 2021, there were 1587 diabetes‐related deaths [5].

Foot complications such as wounds, ulcers, and amputation are a significant risk for people living with diabetes. They result in substantial clinical, social and economic burdens. In 2021, Edmonds et al. [6] reported that with diabetes the lifetime risk of developing a diabetic foot ulcer is between 19% and 34%, and that 85% of diabetic amputations had been preceded by a foot ulcer. The five‐year mortality rate for a person with a diabetic foot ulcer is 2.5 times as high as the risk for an individual with diabetes who does not have a foot ulcer [7]. Lower leg amputations due to diabetes complications are some of the most common operations performed in Fiji, with a significant impact on hospital bed capacity and resourcing [8, 9, 10]. In MICs, diabetic lower leg amputations occur 12 years earlier than in high‐income countries, with a mean age of 58 years [10]. This significantly impacts the workforce [11, 12, 13] and, therefore, Fiji's overall growth and development [13].

Diabetes care is mainly self‐managed, and promoting self‐management adherence is important to prevent the number of complications related to diabetes mellitus. A recent systematic review identified that the key factors affecting self‐management are individual drive, social capital, people's knowledge base and insufficient healthcare [14]. Health promotion is an essential tool to prevent and manage diabetes [15, 16, 17]. Its strategies include empowering people with knowledge of self‐management and creating a supportive environment for people with diabetes, improving community support and ensuring an effective healthcare system that recognises and supports health promotion to manage diabetes effectively [18, 19, 20].

Footcare is a key aspect of diabetes self‐management, critical in avoiding or reducing the impact of foot complications. It includes washing and inspecting feet regularly, checking for foot skin colour and temperature, trimming toenails straight, wearing closed shoes that fit correctly and avoiding walking barefooted to reduce the risk of injury or infection, identifying signs of foot ulcers, wound care and looking for blisters, sores, ulcers, infected corns or ingrown toenails [21, 22].

Fiji lacks the material and human resources to support diabetes care and self‐management fully [23]. This is reflected in the healthcare workforce's inadequate knowledge of diabetic footcare, foot complications, and DSM health promotion [24]. Recent Fijian research found that patients rarely conduct footcare assessments except when they have an infection [8, 10]. There has been a call to resource and train diabetes educators to enhance diabetes self‐management in Fiji and improve attendance at clinics and reduce late presentation of foot complications [10, 24, 25].

Individuals with foot complications can self‐manage their diabetes when provided with the necessary information and resources to do so. Providing foot care and assessment to all individuals with diabetes is essential for all diabetes care services in any healthcare setting to ensure they understand daily management practices, which can assist in recognising early foot complications. The health care services and the health promotional organisations involved need to increase awareness of how foot complications develop and to make known that foot complications leading to amputations are rising; medical information actions are required to integrate into daily diabetes care.

Diabetes‐related foot complications can deteriorate a person's quality of life [26, 27]. Therefore, daily self‐foot assessment and proper self‐management practices such as inspection of feet, minimising the risk of foot complications and professional treatment can help minimise developing foot complications and foot amputations. A collaborative approach is required to treat the condition and prevent associated diabetes foot complications. Literatures revealed that when patients are adequately informed about foot care and the increased rates of amputation, there were improvements in their self‐management practices that reflected improvements in their diabetes [28, 29]. Similarly, improving and providing continuing education is essential for healthcare providers to provide adequate care for those who visit healthcare facilities. According to the American Association of Diabetes Educators, Self‐Care Behaviours framework, people with T2DM should be skilled in self‐care behaviours that improve their quality of life while reducing associated complications of this condition [30].

There appears to be limited research assessing the lived experience of people managing diabetes footcare, with most studies relying on self‐report [31, 32]. Many studies have evaluated the effectiveness of interventions for diabetes footcare self‐management or the effectiveness of the diabetes guidelines [29, 33, 34]. There is some research using wearable cameras to study the risk factors of diabetes, such as physical activity and food consumed [35, 36]. Recently, our research team developed a methodology for studying diabetes self‐management using wearable cameras, such as the Diabetes Cam [37]. This paper uses Diabetes Cam to explore footcare self‐management, its barriers and facilitators and the impact of diabetes foot complications amongst I‐Taukei Fijians (Indigenous Fijians) in Fiji.

## 2 Methods

### 2.1 Study Design

This mixed‐methods research study examines data from the innovative Fijian Diabetes Cam study [37]. The participants wore an automated camera (Autographer Qstarz BT‐Q1300ST) for 4 days on a lanyard around their necks during waking hours. The camera took photographs every 7 s using a 136° lens. The images captured could be analysed for diabetes self‐management behaviours and the participants' environment. Photo‐elicitation interviews were undertaken following the retrieval of the cameras using the images as prompts. Observations were also conducted by KRB of the participants in their home environments during data collection. Data were collected from October 2021 to May 2022, with no COVID restrictions.

Ethical approval was received from participants and the participating facility. The anonymity of participants and third parties was protected by strict data management protocols and by obscuring faces and other identifying features in published images. The research protocol is available at www.otago.ac.nz/heppru/research. Full details of the methodology are published elsewhere [37]. All written material was available in I‐Taukei and English, and participants were advised to respond in whichever language they were comfortable using.

### 2.2 Sampling, Recruitment and Data Collection

We recruited a strategic sample of 30 I‐Taukei adult males and females with a history of diabetes lasting 2 years or longer from each diabetes clinic of the four medical divisions in Fiji. Eligible participants were invited by a letter containing the information for the study, followed by a telephone call. KRB conducted home visits to those expressing interest, obtained consent, and briefed the participants about the study.

Participants were asked to wear a camera for 4 days, from Thursday to Sunday, from waking up until going to bed. They could remove the camera in situations where privacy was expected (e.g., using the toilet or bathroom), if requested, or at any time if they felt uncomfortable. Participants were given information cards to explain the study if other people asked questions or raised concerns. After 4 days, the cameras were retrieved, and all images were downloaded to a password‐protected server without the researcher viewing them. Participants were then asked to view the images and delete any they did not wish the researchers to see. KRB conducted a home visit with the participants to explore the approved images in a photo‐elicitation interview and to complete an observation of the home environment, both of which were guided by a semi‐structured schedule. Key questions included knowledge and practice of footcare self‐management, the impact of foot complications, and the challenges and facilitators of footcare self‐management. The participant or KRB recorded the responses in writing. Researcher observations were also recorded. KRB observed the participants in their homes during the briefing and the review. Demographic data and a brief medical history were also recorded.

### 2.3 Data Analysis

Images were manually coded using content analysis [38], with the image data entered into an Excel spreadsheet using a footcare self‐management data coding protocol (Data S1). The footcare protocol was based on Fiji Diabetes and World Health Organization (WHO) and International Diabetes Federation (IDF) diabetes footcare best‐practice guidelines [21, 22, 39], and a systematic review of diabetes self‐management [14]. Image codes included foot health status, behaviours, and resources. The observations were used to provide further context. Of the 30 interviews, 26 were recorded in English and four in I‐Taukei, translated into English by KRB, then all interviews were transcribed into a Word document. Subsequently, the photo‐elicitation responses were manually coded using content coding to facilitate analysis [40]. Thematic analysis was then employed to analyse the coded data. This involved identifying significant themes or patterns by organising and aligning the codes with potential themes. These themes and subthemes were meticulously reviewed and refined to provide deeper insights into the data [41].

## 3 Results

The findings of the study are presented in four sections: (i) demographic characteristics of the study participants; (ii) analysis of images; (iii) thematic analysis of photo‐elicitation interviews; (iv) the impact of diabetes complications.

### 3.1 Demographic Characteristics

Table 1 describes the demographic characteristics of the study participants. All the participants in the study were over 40 years old, with five (16.7%) being over 60 years old. Almost two‐thirds were female (63.3%). Twenty‐one (70%) were married and nine (30.0%) were widowed. About 17 (56.7%) had received secondary education, while 13 (43.3%) participants had also received tertiary education. Most participants (56.7%) had an average household weekly income of $101 to 200 Fijian Dollars (FJD), four had less (13.3%), and the remaining participants had more (30%). About 24 (80%) participants had diabetes for more than 5 years, and the rest had diabetes for 2 to 5 years.

**TABLE 1:** Characteristics of study participants (*n* = 30).

	Categories	Frequency *n* = 30	Percentage %
Age	40–50 51–60 61–70	13 12 5	43.3 40.0 16.7
Gender	Male Female	11 19	36.7 63.3
Marital status	Married Widow	21 9	70.0 30.0
Level of education	Secondary Tertiary	17 13	56.7 43.3
Weekly income (FJD)	Less than 100 $101–$200 $201–$300 $301–$400 Above $401	4 17 4 2 3	13.3 56.7 13.3 6.7 10.0
Years of having diabetes	2–5 years 6–12 years	6 24	20.0 80.0

### 3.2 Observation of Images

This section describes the results of the content analysis of the images. Regarding the foot health status of patients, of the 30 participants, 10 had visible diabetes foot complications. Five had below‐the‐knee amputations (BKA), and one had a forefoot amputation. Three had an ulcer, and one had multiple unhealed leg wounds.

In relation to diabetes self‐management behaviour, we examined all the images from the 30 participants looking for key aspects of preventive footcare in their daily lives. About 6 of the 30 participants were seen trimming their nails in the images. One participant with a BKA trimmed their toenails after changing the wound dressing. One family member of a participant with an amputation was seen in the images assisting in trimming the toenails. Six participants (two with amputations) and four without foot complications were seen using coconut oil in their footcare. Of the 10 participants who had diabetic foot ulcers or amputations, 4 had unhealed wounds and were seen performing wound care. While the cameras only showed the footwear of four participants without amputations (one wearing flip‐flops and three closed shoes), those with amputations were all seen wearing closed footwear on their remaining foot.

**FIGURE 1:**
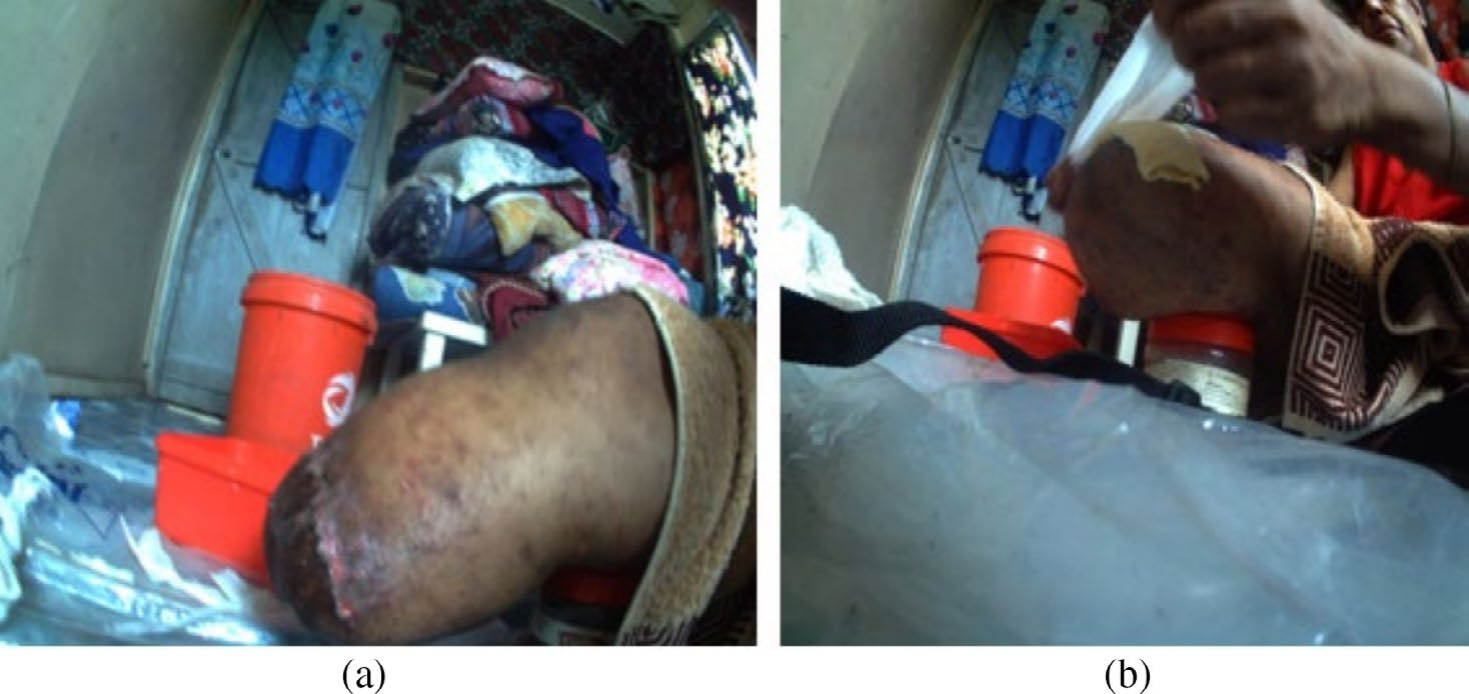
(a) This picture shows a study participant preparing their amputation site for wound dressing. (b) This picture illustrates the participant using reusable materials for wound dressing at home.

We also examined the images for diabetes self‐management resources, including wound care and mobility. Three participants with a BKA utilised unsterilised dressing supplies (cotton wool and gauze rolls) from already open packets (see Figure 1). They also washed their used bandages and hung them out to dry, accessing the washing line outside with assistance from their children and family members. The researcher also observed the dressing kits.

**FIGURE 2:**
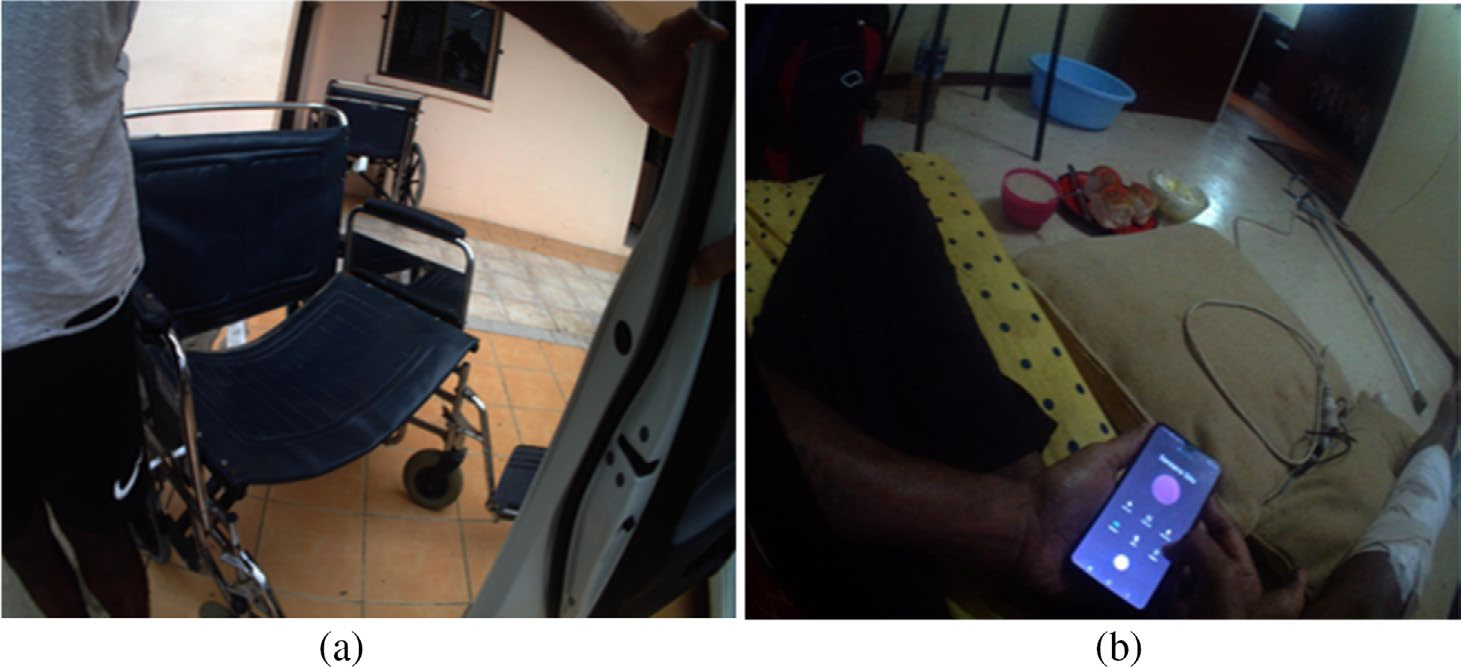
(a) This picture shows a study participant moving from a taxi to a wheelchair at a health clinic. (b) Image depicts a crutch lying by the door and a study participant with multiple leg wounds.

Regarding transport to health clinics, the images showed four participants walking independently and using public transport (bus) or private vehicles. Four of the five people with BKA were seen travelling to the hospital, all needing assistance to transfer from home to a taxi or other carrier (see Figure 2a). Only two of the five with BKA amputations had a wheelchair. The other three were observed crawling around the house. Only two of the three with foot ulcers had a walking stick; the person with multiple leg wounds had one crutch (see Figure 2b), and the person with the forefoot amputation had a four‐legged walking stick.

**FIGURE 3:**
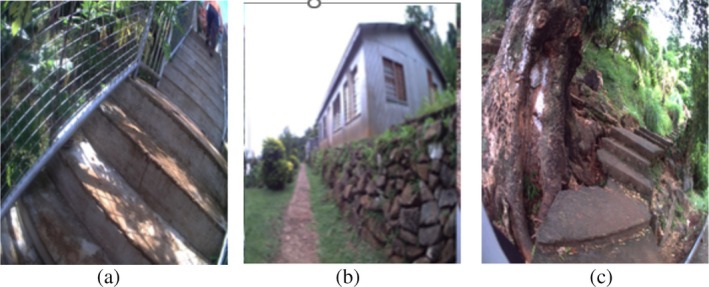
(a) Image shows steep steps outside leading to a participant's house. (b) A long walkway outside is shown leading to a house. (c) Steep steps that can be seen on the slope.

**FIGURE 4:**
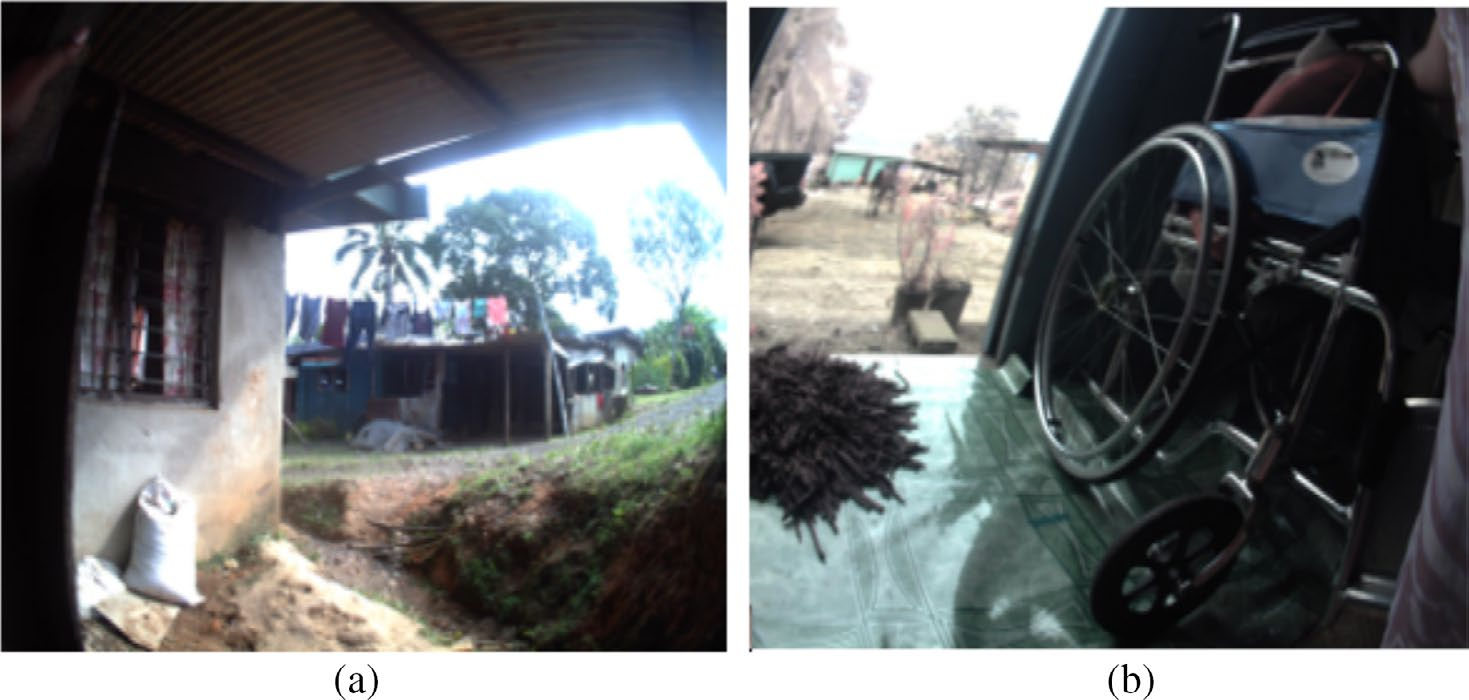
(a) This picture shows inadequate access to the house. (b) The image shows that the participant uses a wheelchair, but the house has no ramp.

None of the 10 participants with foot complications had adequate access to their house (see Figure 3a–c). The participant with a forefoot amputation used a four‐legged walking stick to walk up and down the steep steps at home (see Figure 3a). Participants with amputations who used wheelchairs had limited access in and around the house. For them, furniture was moved to the edges of the living room to allow space to manoeuvre the wheelchair. Neither participant had a wheelchair‐accessible bathroom. They both braced themselves against the wall and hopped. Furthermore, they did not have access outside due to uneven surfaces from the house unless assisted by family members (see Figure 4).

### 3.3 Analysis of Photo Elicitation Interviews

#### 3.3.1 Footcare Assessment Practices

All participants indicated they did some form of footcare. Most (19) participants said they looked for cuts and wounds, and a common practice was to check their feet during or after a bath.

I look for any cut or sore after a bath. (P2, female 63 years)

Before I wear my shoes, I will check for any cuts or blisters. (P3, female 44 years)

Twenty‐five participants said they applied coconut oil on their feet after a bath, and some looked for cuts and wounds at this time.

I apply coconut oil every day after my bath, and that's when I look at my legs and feet. (P7, male 54 years)

#### 3.3.2 Knowledge of Footcare Assessment

When asked, ‘Do you know how to perform a foot assessment?’ 19 participants knew how to look for cuts and wounds to reduce the risk of amputation. The others did not comment.

I do not know the details of doing a foot assessment and what to do. (P18, female 49 years)

Participants were asked, ‘Do you know how to trim toenails, and why do you need to trim toenails?’ Eighteen participants said they trimmed toenails when they grew long but did not see that it was as part of diabetes footcare. Twenty‐seven participants were not aware of the recommendation that toenails be trimmed flat.

I cut my toenail short so that it is clean because if it is long, it can make me trip over on an object. (P17, female 55 years)

#### 3.3.3 Addressing Potential Foot Complications

Twenty‐two participants said that if they sustained a new wound or experienced any foot problem, they visited the clinic as soon as possible. The others did not comment.

… I do not want to get my leg amputated. It will make life more difficult. (P21, male 55 years)

#### 3.3.4 Footwear Knowledge

When asked about shoes for diabetes foot care, 16 participants responded that they did not know the importance of selecting appropriate shoes.

I wear my shoes, which are comfortable. I don't know what shoes are good for us. (P13, female 50 years)

I … wear slippers because it is hot, and … comfortable …. (P23, male 52 years)

The 10 participants with foot complications stated that during their hospitalisation or at the footcare clinic, the nurse or the doctor advised them to wear closed shoes that were comfortable and well‐fitted.

The doctor said I should wear soft and comfortable shoes. (P24, female 55 years)

Some participants noted that specific jobs required wearing boots and closed hard shoes as part of the uniform, which could be a concern.

I started the blister on my foot because of my boots, the working shoes. (P14, male 48 years)

#### 3.3.5 Provision of Footcare Health Information

Twenty‐six participants said they were given brief instructions on what to do with their feet if they sustained a wound, that is, to visit their health provider at the earliest time. The others did not comment.

If they tell us how to do foot assessment, then I will do it that way; all they say is watch your feet; if any cut, bring them to the hospital faster for them to see. (P6, female 62 years)

Eighteen participants indicated they did not receive adequate information on conducting foot care.

They never tell me any details about assessing the feet. (P12, female 47 years)

#### 3.3.6 Fear of Amputation

Six participants without amputations discussed their Fear of amputation.

I'm afraid to have my leg cut. (P1, male 46 years)

I am afraid of getting a foot amputation. My mother had an amputation, which led to her death. (P16, male 60 years)

I refuse to have an amputation … I know that diabetes makes you lose your leg, so I am trying to save my leg to the best possible I can. (P14, male 48 years)

### 3.4 The Impact of Diabetes Complications

#### 3.4.1 Wound Care

The 10 participants with amputations or wounds said they needed to visit the health clinic daily for wound dressing; however, they all faced significant barriers. Interviewees said it was frequently not possible due to the expense of catching a taxi and because they required assistance from family to get into the cab. Furthermore, wound dressing materials were unavailable if they could not access the clinic for daily dressing; no alternative provisions were available.

Sometimes dressing supplies will be given but very little. I have to buy my supplies for home use, and sometimes, I rewash the used ones for the next use. (P12, female 47 years)

All 10 participants with foot complications said they did not have enough dressing supplies, and these were expensive to purchase, and 8 said they washed their dressings to reuse them.

I use diapers to dress my wound at home. (P14, male 48 years)

Four participants said they were advised to add salt to regular tap water to clean their wounds.

#### 3.4.2 Mobility Difficulty

Patients with an amputation found this decreased their mobility and ability to participate in activities of daily living, and this had a significant impact on them.

I crawl around in the house. (P10, female 55 years)

I have been given crutches, but at my age [it] was difficult to use these crutches. (P12, female 47 years)

**FIGURE 5:**
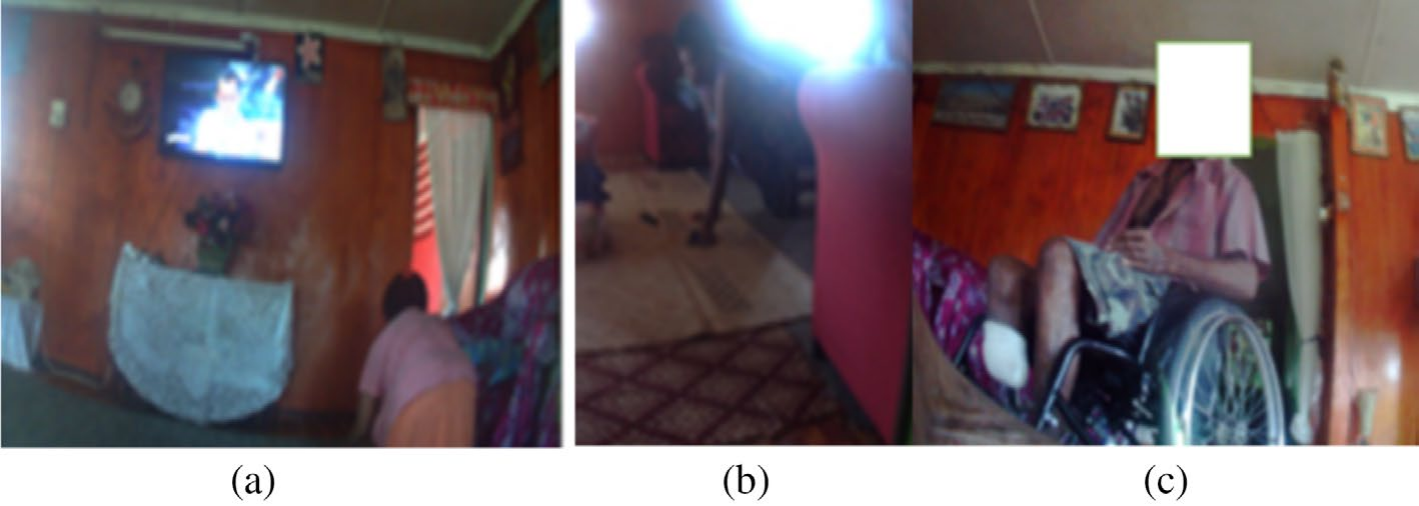
(a and b) Image shows a participant crawling around the house. (c) Image shows a participant sitting in a wheelchair in his home.

Figure 5a,b shows a participant crawling around in the house; they explained that a wheelchair was too expensive and that she could not afford one. Another participant used an office chair to move around the house and do housework and activities of daily living.

I use the office chair to help me move around in the house doing my household chores. It was expensive to buy a wheelchair. (P12, female 47 years)

#### 3.4.3 Transportation to the Diabetes Clinic

Most participants said they had difficulty accessing health facilities as they could not travel on public transport. Those with amputations said they sometimes missed their clinic due to difficulty accessing transport.

My husband will assist in holding me or even sometimes lift me from home to the roadside, where I will take a taxi to the hospital for about $15 a day. … I use a wheelchair to move around. (P12, female 47 years)

I' … travelled to a health clinic in the truck. The drive is about $50. There are no bus services in this area. (P15, female 58 years)

#### 3.4.4 Withdrawal From Social Events and Family Activities

The participants who had amputations were not able to attend social activities outside the home due to mobility difficulties and the poor availability of mobility aids. One participant said they do not like to trouble their family and loved ones.

I have to stay home most of the time because my relatives need to enjoy themselves at family gatherings, such as church service. [It is] tough to move around with no proper walking aids. (P10, female 55 years)

#### 3.4.5 Loss of Employment

Foot wounds or amputation impacted people's ability to work. Five participants had to leave paid employment. Reasons included the disease itself, the need for frequent absences, the time involved in attending clinics, the lack of mobility aid, and the inability to get transport to work.

I have been on medical leave from my work … the doctor requires me to go to the specialized hospital for further investigation …. (P14, male 48 years)

I had my left leg amputated two years ago. Diabetes makes you lose your leg, lose your job, and it is tough to live with. (P10, female 55 years)

The financial constraints from unemployment create additional financial difficulties and compromise patients' diabetes self‐management practices, such as buying footcare supplies.

### 3.5 Facilitators and Barriers to Living With Diabetes

#### 3.5.1 Family Support

**FIGURE 6:**
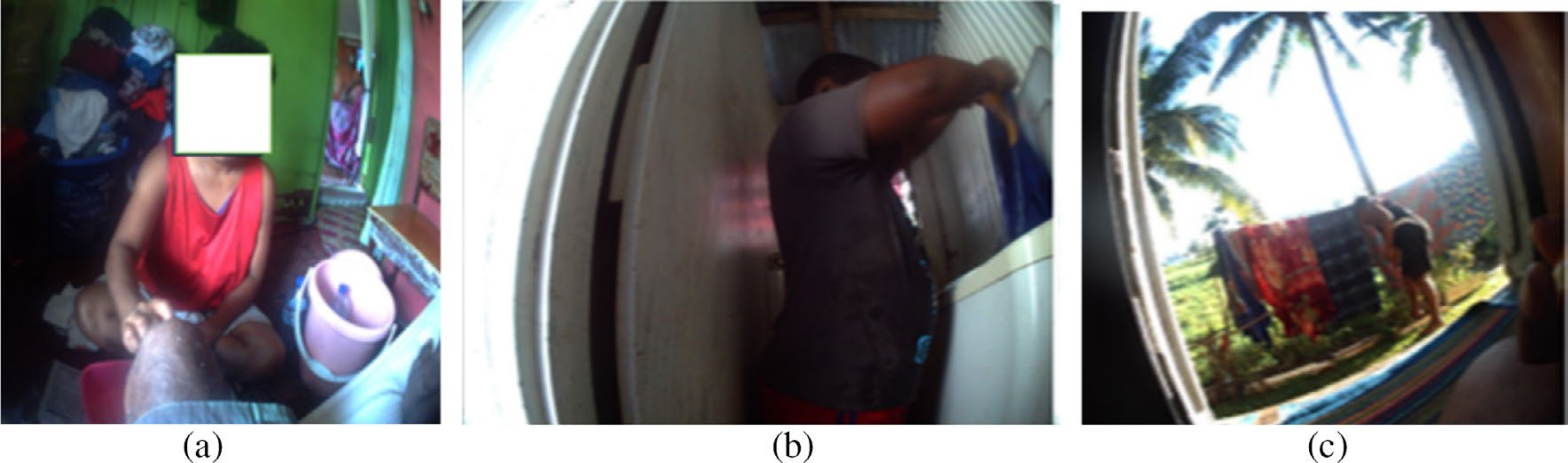
(a) Image shows a daughter performing wound care for her father with a BKA. (b) This picture shows a participant's son assisting in washing the clothes, supervised by his mother, who has a BKA. (c) Image showing a participant's son hanging the clothes outside on the line, supervised by his mother, who has a BKA.

When asked, participants identified family support as a crucial facilitator, especially for people with amputations. They required assistance from family to help with their daily activities, such as meal preparation, foot assessment, wound care (see Figure 6a), household chores (see Figure 6b,c), mobility, medication administration and being a support person at healthcare appointments.

Lack of social support was a barrier for participants with amputations, as highlighted by one participant.

It is a tough life for me. Sometimes I don't eat because nobody prepares my food. I will wait for my daughter after work, then we will eat. Then I will take my medicine. (P10, female 55 years)

#### 3.5.2 Community and Religious Social Groups

Community and religious social groups also facilitate support for diabetes self‐management with a focus on sharing health information.

I am involved in the women's group in my village, and we always invite the nurses and the health workers to give talks on healthy living, like diabetes. In our church program ….(P17, female 55 years)

#### 3.5.3 Lack of Resources

The participants lacked the necessary resources for diabetes self‐management, including basic diabetes control. When asked about using blood sugar testing kits, participants were aware of the need to stabilise their blood sugars, yet none had a blood sugar testing kit, and 29 participants could not afford one.

I don't have a machine to check my sugar. It is costly to buy as it is about $100. (P10, female 55 years)

Three participants discussed using herbal medicines when they had no available medicines and dressing supplies.

When my medication's finished, I drink some herbal medicines to keep me going until I have money to buy my medicine. (P17, female 55 years)

I applied traditional leaves to my wound when I didn't have ointment. (P10, female 55 years)

#### 3.5.4 Mobility Aids

With the unavailability of mobility aids, a woman with a below‐knee amputation used an old five‐legged office chair to move around the house doing household chores, navigating with a good leg. A participant with below knee amputation as in Figure 5c uses a wheelchair to move around in the house, household furniture's were moved to the side of the house to allow accessibility. Another woman with a BKA who a wheelchair does not have had to crawl around the house for activities of daily living.

#### 3.5.5 Climate Conditions

Participants said they were used to being barefoot inside the house and preferred to use flip‐flops or slip‐on shoes when going out because Fiji was hot and they were more comfortable than covered shoes.

I wear flip‐flops because I feel good, and it is very hot weather; my leg usually sweats with shoes. (P17, female 55 years)

#### 3.5.6 Knowledge From Advertisements and News Items

Four participants stated that advertisements and news items about diabetes complications had raised their knowledge of diabetes symptoms and management.

They will send me to see the dietician. I will be waiting, and when it takes longer, I will go from there and start searching on Facebook what good food for diabetes. (P5, female 55 years)

I hear news from the TV and radio …. (P6, female 62 years)

#### 3.5.7 Footcare at the Health Clinic

When participants with foot complications can access the health clinic for foot care, appropriate care is given, as one participant noted.

I had my toes amputated about a year ago. It started as a blister, which I was unaware of until I saw discharges coming off my toes … I went to the hospital, and the doctor told me that my sugar was very high. (P25, female 44 years)

#### 3.5.8 Public Health Screening Events

Some participants who go to work and have limited time to wait for their health clinic appointments tend to wait and check their blood sugar levels at periodic health screening programmes nationwide. These events occur regularly at significant events in Fiji and include health education, coaching and diabetes testing.

… When I hear or see the nurses conducting the screening at the parks or any place I will go, that is a good opportunity to check my blood sugar because it is always empty. (P3, female 44 years)

## 4 Discussion

This study explored the footcare self‐management of I‐Taukei patients with diabetes in Fiji. While all participants said they performed footcare, for most, this was limited to looking for cuts and wounds as that was all they had been advised to do by their health providers. In the interviews, many participants noted frustration with a lack of detailed educational advice. Many participants also applied coconut oil to their feet after a bath, which provides an opportunity for foot assessment if people know how to do it. The majority of participants knew that if they had a new wound, they should go to the clinic as soon as possible. This finding about the lack of footcare is consistent with previous Fijian research [8, 10] This study identifies significant gaps in health promotion, such as building the personal skills of individuals with diabetes to perform foot assessment and recognising the development of foot complications. Showcasing and demonstrating how to perform foot assessment in every healthcare clinic is essential to equip individuals with diabetes to acquire the skills to perform foot assessment and foot examination. Using a checklist that contains required activities such as monitoring blood glucose levels, adhering to a healthy diet, engaging in regular physical activity, taking medications and attending regular check‐ups plays a vital role in maintaining health and well‐being for every diabetes patient that attends the clinic to improve life in support of diabetes self‐management.

Unsurprisingly, the images detected few foot care practices except those who had already developed foot complications. Trimming nails was seen six times in the images, and footwear was rarely seen except for those with amputations. Over half of the participants did not know the importance of selecting appropriate shoes. However, all 10 participants with foot complications had received comprehensive footcare advice at the clinic, a finding found elsewhere [42].

Our study suggests that while footcare self‐management education is very limited in Fiji, people are provided with better advice once complications result or amputations occur. It also indicates that people recognise the need for self‐management of footcare, and that they largely do as instructed. A recent systematic review found that patient motivation for self‐management was compromised by the quality and inconsistency of information delivered by health professionals [14]. It seems likely that if comprehensive advice was provided, with appropriate support and resourcing for preventive footcare, people may follow it, especially given the awareness and Fear of amputation. The lack of focus on preventive footcare in the health system, including the lack of more detailed self‐care information, resources, and support before foot complications occur, is problematic given the literature shows that these health promotion activities can lower diabetes complication scores [43, 44]. However, our findings are consistent with previous research in Fiji that found healthcare workers lacked this knowledge and that there was a lack of human and other resources to support diabetes self‐management [13, 14].

A third of the study participants had diabetes foot‐related amputations or foot ulcers, in keeping with the extent of diabetes‐related amputations in Fiji [8, 10]. These participants experienced difficulty accessing health care services due to transportation issues, and they sometimes missed clinics. Researchers suggest that missed clinics hinder patient compliance and are a critical element in the success or failure of treatment [45]. Further, those with foot complications left work due to their diabetes, the time involved in self‐management, and the lack of support they received. These findings are consistent with research from other countries that showed the impact of diabetes complications on job losses [11, 12, 46, 47]. They also underscore the early age at which people require amputation in MIC [10], and, therefore, the impact on the economy.

This study revealed the need for supportive environments to ensure that individuals with diabetes‐related amputations could perform the required care for managing diabetes, such as physical activity and attending community access and social visitations. The inclusion of community development to support diabetes care is therefore warranted.

In exploring other facilitators and barriers to self‐management, family support was identified as a key facilitator, particularly for those with amputations, in line with previous research [14]. Without this support, it can be a ‘tough life’, as one participant with an amputation noted. Community and church support was also important to participants in managing their diabetes [14]. Key barriers were the lack of dressing supplies, blood sugar testing kits, and mobility aids—one woman with an amputation innovated using an office chair to get around her house. Previous research has indicated that proper wound dressings, ointments and supplies promote diabetes foot ulcer healing, reduce the cost of treating diabetes foot ulcers and reduce patient pain [48]. The lack of such supplies in this study resulted in some participants using herbal medicines. The warm climate made wearing closed shoes complicated, likely an issue across the Pacific and other LMICs. Despite gaps in care, participants reported receiving appropriate footcare when they had complications and could access clinics [14, 49].

As the right to health for every individual, people with diabetes also demand the need to be provided with mobility aids and dressing supplies for their wounds [50, 51], to assist them in attending health care clinics to monitor their disease.

People with diabetes foot amputations are faced with difficulties accessing healthcare clinics due to transportation issues; they require a healthcare system approach to deliver health promotional activities such as community outreach screenings and house visitation and strengthening of domiciliary visits for those with diabetes‐related disabilities, Provision of supportive Transportation to bring them into healthcare settings for detailed investigations and health checks.

Environmental factors also impacted, such as lack of access in and out of their homes, which created demonstrable daily hardships, particularly for those with amputations, and were visible in the images and observation [25, 52]. Housing is a social determinant of health. Existing research suggests that constrained accessibility around the house poses challenges to self‐management in daily living and to patient well‐being [53, 54]. The WHO housing guidelines report that adequate accessibility should be considered in the design of the indoor and outdoor housing environment, especially for those with a disability; accessibility and wider housing quality are essential for those with diabetes and complications such as amputations and can help to reduce other potential health risks. Researchers have identified that poor‐quality construction or poorly maintained houses can increase the risk of fall‐related injury [53, 55].

## 5 Strengths and Limitations

To the best of our knowledge, this is the first time that wearable cameras have been used alongside photo‐elicitation and observation to study diabetes self‐management. Wearable cameras are a strength as they enable an objective view of the participant's behaviour and environment, facilitate the photo‐elicitation conversation, overcome recall bias and inform the researcher, who could then engage in more in‐depth inquiry with the participant. Unlike the role of wearable cameras in studying the food environment [36], footcare was not often detected in the images; footcare practices were either infrequent or were behaviours that might only occur once a day, possibly when people had removed the camera for bathing. However, the wearable cameras were much more valuable for those with foot complications as they captured many more relevant images and provided more insights into the challenges of daily living.

This study provides valuable insights into the diabetic footcare self‐management practices of I‐Taukei Fijians. While the study used a strategic sample and provided insights across Fiji, the results may under‐report the extent of the challenges facing I‐Taukei patients with diabetes. Despite being a small study from Fiji, this research provides insights into diabetes footcare self‐management that may be valuable for other Pacific nations and countries battling diabetes. Image analysis, observation and photo‐elicitation provided richer data than any method on its own.

## 6 Implications of the Study

This study can be used for advocating for systemic change and wider health promotion actions to prevent diabetes and diabetic complications. Recognising the current high demand on the health system from diabetic complications, there is also an urgent need to reorient the health system to prioritise health promotion and preventive care for people with diabetes, and for more community‐based resources and support for patients with diabetes. This may include workforce training and development, improving access to health services, co‐designing patient‐centred services to promote engagement and attendance and planning and designing evidence‐based footcare management systems, checklists and guidelines for footcare services and diabetes clinics in Fiji.

This study highlights the lack of healthcare services and support for effectively assisting people to manage their diabetes footcare and the challenges facing an underfunded and under‐resourced system that will increasingly be required to address the rising rates of diabetes. While improved economic prosperity in Fiji may improve the situation over the longer term, there is an urgent need for donor countries and WHO to strengthen their support for managing such an impactful health promotion issue in Fiji and other LMICs.

This study also highlights the need for wider, multi‐sectoral health promotion action in Fiji. Our study suggests that community‐based assessment and modification of housing should be funded and included as part of a wider healthcare response to enable improved mobility and reduce further complications for people with diabetes. The study identifies the need for adequate income support and disability support to facilitate diabetes self‐management, as well as employment and building regulations to enable those with amputations and severe foot complications to participate in society and remain in the workforce if appropriate. This paper demonstrates the value of understanding the lived experience of diabetes footcare self‐management. Further research is needed in Fiji to explore other aspects of diabetes self‐management. This research method may be valuable for studying diabetes self‐management in different countries.

## 7 Conclusions

Effective diabetes footcare self‐management contributes to better diabetes care and can prevent diabetic amputations and enhance lives. Yet, there were numerous barriers for I‐Taukei patients in Fiji. Despite the importance of footcare in the diabetes management guidelines, the health system is not currently oriented toward health promotion and prevention. As a result, patients remain unsure and largely unsupported unless they have already developed complications. Patients could not easily access essential health services and information because of a lack of transportation or community‐based options to access health care and a lack of workforce capacity for preventive care. Further, there was a lack of community‐based support and basic supplies and resources for self‐management, such as wound dressings, and the physical housing environment was disabling for those with complications. Healthcare funding and broader support for patients with diabetes in Fiji is currently inadequate, and there is a pressing need for the Fiji government, donor countries and WHO to strengthen preventive action. There are presently many missed opportunities to improve diabetes self‐management to reduce the rate of amputations and promote the health of people with diabetes in Fiji.

## Author Contributions

**Keresi Rokorua Bako:** conceptualization, investigation, methodology, data curation, formal analysis, writing – draft preparation and project administration. **Masoud Mohammadnezhad:** conceptualization, methodology, writing – review and editing, Supervision. **Dianne Sika‐Paotonu:** conceptualization, writing – review and editing, supervision. **Amanda D'Souza:** analysis, writing – review and editing, supervision. **Louise Signal:** conceptualization, methodology, validation, formal analysis, writing – review and editing, supervision.

## Ethics Statement

The study received ethics approval from study participants and participating facilities. All participants were required to read an information sheet and consent by signing the consent form before being recruited to wear the cameras and participate in the photo‐elicitation process.

## Conflicts of Interest

An Otago University Pacific PhD Scholarship funded Keresi Rokorua Bako. Masoud Mohammadnezhad is a of the Faculty of Health, Education, and Life Sciences, Birmingham City University, Birmingham, UK. Dianne Sika‐Paotonu, Amanda D'Souza and Louise Signal is faculty members at the University of Otago. Louise is an editorial board member of HPJA, we acknowledged that she will be excluded from all editorial decision‐making related to the acceptance of this article for publication.

## Supporting information


Data S1.

## Data Availability

The data that supports the findings of this study are available in the Supporting Information of this article.
