# Supplementary material for: ‘Diabetes Makes You Lose Your Leg’: Footcare Self‐Management Amongst I‐Taukei Fijians—A Wearable Camera Study
Source: Health Promot J Austr. 2025 Jul 10;36(3):e70076. doi: 10.1002/hpja.70076 (PMC12246285; doi:10.1002/hpja.70076)
Supplement: Supplementary file 1 — Data S1. [file HPJA-36-0-s001.docx]

*Department of Public Health, Health Promotion and Policy Research Unit*

*University of Otago, Wellington*

*New Zealand*

**Supplementary File 1:**

**Diabetes Cam: Footcare Self-Management And The Impact Of Diabetes Foot Amputations In Fiji – Coding Protocol**


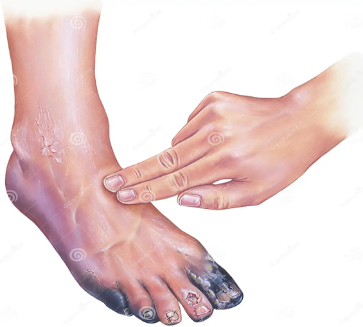


Bako KR, Mohammadnezhad M, Sika-Paotonu D, D’Souza A, Signal L.

*Citation: Bako KR, Mohammadnezhad M, Sika-Paotonu D,* *D'Souza A, Signal L. Diabetes Cam: Footcare self-management and the impact of diabetes foot amputations - Coding Protocol*

*Health Promotion Policy and Research Unit, University of Otago, Wellington, New Zealand.*

This coding protocol was developed to guide the coding of the footcare activities in the images and observations of the participant’s environment in the Diabetes Cam Fiji ^1^. The protocol was based on appropriate diabetes guidelines as outlined below.

**Coding Ethics**

The ethical considerations in coding the images are outlined below:

1. The anonymity of all participants, third parties, and their environments is protected.

2. The privacy of those who may be inadvertently captured in the image is protected.

3. All images used in the disseminated material with identifiable people, street names, places, shops, and business names must be blurred.

4. The demographic information collected will only be viewed by the core Diabetes’ Cam research team.

5. The data must be kept on secure University of Otago computers that are password protected.

6. During the data analysis, the equipment will never be left unattended.

7. If the researcher leaves their computer for any amount of time, they must log out.

**Footcare codes and definitions**

| **Code** | **Definition** |
| --- | --- |
| Diabetic foot ulcer present | A diabetic foot ulcer is an open sore or wound that occurs in patients with diabetes, and is commonly located on the bottom of the foot ^2^ |
| Amputation | Amputation is surgery to remove all or part of a limb or extremity ^2^ |
| Wound care | The management of diabetic wound requires offloading the wound, daily saline or similar dressings to provide a moist wound environment ^2, 3^ |
| Trim toenails | Trim your toenails straight across and gently smooth any sharp edges with a nail file ^2-4^ |
| Footwear selection | Wear shoes that fit well ^2, 3^ |
| Mobility aids | Crutches, walking stick, wheelchair, other aid ^3, 4^ |
| Physical home environment for people with diabetes | Adequate housing that is structurally sound, and that provides sufficient space and protection against other threats to health, and which allows adequate mobility and access to the activities of daily living  An optimal physical home environment includes adequate mobility access for those with walking aids ^5^ |
| Transportation | Car, bus, van, taxi, carrier |

**Coding Spreadsheet**

| ***Identification*** | | ***Health status*** | | ***Behaviour*** | | | ***Resources*** | | | | |
| --- | --- | --- | --- | --- | --- | --- | --- | --- | --- | --- | --- |
| ***Partn***  ***ID*** | ***Image ID*** | ***Diabetic foot ulcer present***  ***(Y/N)*** | ***Amputati (Y/N)*** | ***Wound care***  ***(Y/N)*** | **Trim toenails**  ***(Y/N)*** | ***Footwear***  ***(Describe)*** | **Mobility aids**  **(Describe)** | ***In-house physical layout***  ***(Describe)*** | ***Access to the house\in and out***  ***(Describe)*** | ***Transportation***  ***(Describe)*** | ***Comments*** |
| ***001*** |  |  |  |  |  |  |  |  |  |  |  |
| ***002*** |  |  |  |  |  |  |  |  |  |  |  |
| ***003*** |  |  |  |  |  |  |  |  |  |  |  |

**References**

**1.** Bako KR, Mohammadnezhad M, Sika-Paotonu D, Sime S, Signal L. Diabetes Cam: An Objective Methodology to Study Diabetes Self-Management. *American Journal of Preventive Medicine.* 2023/12/22/ 2023.

**2.** World Health Organization. *Prevention, Identification, and Management of Foot Complications in Diabetes* April 2011.

**3.** Fiji Ministry of Health & Medical Services. *Diabetes Guidelines and Footcare* <https://www.health.gov.fj> 2012.

**4.** Bakker K, Apelqvist J, Lipsky B, Van Netten J. International Working Group on the Diabetic Foot. The 2015 IWGDF guidance documents on prevention and management of foot problems in diabetes: development of an evidence-based global consensus. *Diabetes Metab Res Rev.* 2016;32(Suppl 1):2-6.

**5.** World Health Organization. WHO Housing and Health guidelines. 2018.
